# Challenges Faced by People Living with HIV/AIDS in Cape Town, South Africa: Issues for Group Risk Reduction Interventions

**DOI:** 10.1155/2010/420270

**Published:** 2010-09-02

**Authors:** Allanise Cloete, Anna Strebel, Leickness Simbayi, Brian van Wyk, Nomvo Henda, Ayanda Nqeketo

**Affiliations:** Social Aspects of HIV/AIDS and Health, Human Sciences Research Council, Cape Town 8000, South Africa

## Abstract

This paper presents the findings of an exploratory study to investigate the challenges faced by people living with HIV/AIDS (PLWHA) in communities in Cape Town, South Africa. The primary goal of the study was to gather data to inform the adaptation of a group risk reduction intervention to the South African context. Qualitative methods were used to examine the experiences of PLWHA. Eight focus group discussions (FGDs) were conducted with 83 HIV-positive participants and 14 key informants (KIs) involved in work with PLWHA were interviewed. Findings revealed that AIDS-related stigma was still pervasive in local communities. This was associated with the difficulty of disclosure of their status for fear of rejection. Also notable was the role of risky behaviours such as lack of condom use and that PLWHA considered their HIV/AIDS status as secondary to daily life stressors like poverty, unemployment, and gender-based violence. These findings have implications for the adaptation or development of behavioural risk reduction interventions for PLWHA.

## 1. Introduction

As people living with HIV/AIDS (PLWHA) now live longer and healthier lives due to the greater availability of antiretroviral (ARV) treatment, the urgency of including behaviour change strategies for PLWHA into the public health system becomes an imperative to curb the further spread of the disease and also to prevent reinfection [1]. Moreover, where ARV treatment is readily available decreasing HIV/AIDS morbidity has been paralleled by increasing HIV/AIDS infection rates suggesting that prevention programmes targeting only HIV-negative persons may be inadequate to curb the HIV epidemics [2]. 

Behaviour change strategies tailored to the specific needs of PLWHA are termed positive prevention. In the United States of America (USA), positive prevention is now the standard for HIV prevention and many of the intervention models that have shown efficacy in reducing unsafe behaviour are being replicated in South Africa [3]. South Africa is currently estimated to have amongst the most PLWHA globally [4]. Addressing the HIV/AIDS prevention needs of PLWHA becomes even more of a priority in the light of studies illustrating that a significant minority of PLWHA in South Africa are still engaging in risky behaviour [5–8]. Hence, a public health imperative is created to address the HIV risk reduction needs of PLWHA. 

Risk reduction interventions designed to reduce HIV transmission risk behaviours in people who know they are HIV-positive have demonstrated risk reduction benefits in several studies conducted in the USA [9–13]. Within African contexts, the massive roll out of ARV treatment, presented an excellent opportunity for healthcare providers to link prevention with care for PLWHA. A risk reduction intervention, known as Options for Health, is one of the first USA-developed interventions [14] which was adapted in South Africa and shown to be efficacious in reducing the HIV risk behaviour of HIV-positive people on ARV treatment in Durban, KwaZulu-Natal (KZN), South Africa [15]. Whilst Options for Health had promising results, other interventions that can be delivered in the group format, thus impacting more people at one time than the individually administered Options for Health intervention should also be tested. Indeed, there is one evidence-based risk reduction intervention which was originally developed in the USA known as Healthy Relationships [16, 17]. Healthy Relationships, is suitable for small groups and is a support group-based intervention that assists PLWHA to manage AIDS stigma, make effective HIV status disclosure decisions, and practice lifelong safer sex. The intervention demonstrated evidence for efficacy in a randomized controlled trial (RCT) conducted in the USA [16, 17]. Subsequently the USA's Centers for Disease Control and Prevention (CDC) developed Healthy Relationships as part of its Dissemination of Effective Behavioral Interventions (DEBI) initiative. Healthy Relationships has now been implemented in more than 300 agencies in the USA.

The intervention is broadly based on social cognitive theory. In its original form it was administered in five 3-hour sessions. The programme is designed to teach some generic skills which are used to help reduce participants' stress related to safer sexual behaviours and disclosure of their serostatus to family, friends, and sexual partners. The intervention is highly interactive and promotes discussion between group members by using modelling, role-play, and feedback to teach and practice skills (e.g., problem-solving and assertive listening) related to coping with stress; providing personal feedback reports to motivate behaviour change and the use of movie clips to set up scenarios about disclosure and risk reduction to stimulate discussions and role-plays [16]. Six group facilitators with a minimum requirement of a basic social science degree with previous experience of counselling and/or group facilitation were recruited. Group facilitators underwent a five-day intensive training session where each day was spent on each intervention session. Training consisted of primarily role plays of each session and was facilitated by a counsellor, who was trained by the developer of the intervention. 

Since Healthy Relationships was originally developed for use in the USA, some of the intervention activities (movie clips, role plays, risk reduction activities) might not be contextually relevant and appropriate for use with PLWHA in Southern African countries. Our research team working together with its partners in the Social Aspects of HIV/AIDS Research Alliance (SAHARA) collected formative data to culturally adapt Healthy Relationships for use in Botswana, Mozambique, Lesotho, and South Africa. Understanding the contextual factors that influence risk-taking behaviour is important since it is within this social context that PLWHA manage risk taking behaviour, negotiate for safer sex, and navigate their everyday lives. Hence formative research was used to explore the social contexts of PLWHA living in Cape Town, South Africa. The findings from this study were used to primarily inform the cultural adaptation of the major intervention activities like the movie clips, role plays, and risk reduction activities to Southern African context. This phase of the study was the first part of a larger research project to evaluate the efficacy of a culturally adapted Healthy Relationships risk reduction intervention in Southern Africa.

## 2. Methodology

A qualitative study was conducted as part of formative research for the cultural adaptation of Healthy Relationships to the Southern African context. For this study, 14 key informants (KIs) were interviewed, seven of whom were affiliated to support groups for PLWHA and others, and the other seven represented nongovernmental organisations (NGOs), activist groups, government departments, or training services. In addition, eight focus group discussions (FGDs) were held with existing support group structures that are situated at either ARV sites or organisations that provide services to PLWHA. Access to these support groups was obtained through some of the key informant participants. Focus group participants were 18 years and older, HIV-positive, and belonged to already existing support groups for PLWHA. Three mixed-sex and five single-sex groups participated in the FGDs. The total number of participants for these focus groups was 83. Group participants were primarily recruited from support groups based in working class communities and townships in and around Cape Town. Focus groups consisted of men (both heterosexual and same sex) and women from mostly black African and mixed race sociocultural backgrounds in nontreatment linked support groups, men and women from support groups of PLWHA who are on ARV treatment, a white, gay men's support group, and pregnant women from the Mothers-to-Mothers-to-Baby (M2M2B) support groups. Demographic information was not collected from focus group participants.

KI interviews and FGDs were conducted by PLWHA who are open about their HIV-positive status. Interviewers were trained in qualitative interviewing skills and in facilitation of group discussions. KI interviewers and group facilitators followed an interview guide. Appointments were made with KIs and interviews were held at venues suitable for KIs. FGDs were held at the venues where the participants usually met for their support group meetings. Group discussions were conducted in the language of choice of the participants. Issues explored in both the individual and group interviews included the challenges facing PLWHA as well as their experiences of the value of and need for support groups. KI interviews and FGDs were audio-recorded. The audio tapes were then transcribed verbatim, and translated where necessary.

Data were analysed using thematic analysis. The following steps were taken to analyse the data: transcripts were coded using the participants' own words and phrases and without preconceived classification; the participants' language or phrases were examined, categorised, and recurrent themes were identified. Recurrent themes are the similar and consistent ways people think about, and give accounts concerning particular issues [18]. Examples of repetition, explanation, justification, and vernacular terms were highlighted. These were then coded with a key word or phrase that captured the essence of the content, and were taken to constitute emergent themes [19, 20]. 

All participants in this study took part voluntarily and anonymously, and signed consent forms beforehand. Ethical clearance was obtained from the Human Sciences Research Council's Research Ethics Council (REC 3/13/10/04).

## 3. Results and Discussion

### 3.1. Challenges for PLWHA

The main challenges faced by PLWHA, as identified by KIs and focus group participants, related to (i) HIV/AIDS-related stigma and “othering” (blaming and stigmatising the “other” whereas the “other” is defined as someone with a different religion, ethnic group to one's own, and gay men) for the spread of HIV; (ii) disclosure of HIV status to others; (iii) risky behaviours for HIV infection; (iv) the importance of the socioeconomic context within which people with HIV live.

#### 3.1.1. HIV/AIDS-Related Stigma and “Othering”

A dominant theme in this study was the extent to which stigma and discrimination remained a central concern for PLWHA:

*“To me stigma still exists a lot and it will never perish, because maybe if you can get sick and lose weight, people will point fingers at you even if you are not HIV-positive, and they will say that you have AIDS.” *(FGD—HIV-positive African women)

*“The problem that we are facing as a person living with this virus, there's a stigma and a discrimination, because people, if you say ‘I am HIV-positive', they are going to chase you away.”* (FGD—HIV-positive African men and women)

*“When it comes to our [people of mixed race], you know it's funny, our [people of mixed race] have a different mentality in a way, because when it comes to the HIV thingy, our [people of mixed race] don't want to get involved too much, or associated with HIV and AIDS, because there's still very much stigma and ignorance.” *(KI—Community AIDS counsellor)

Reluctance and anxiety to disclose HIV-positive status, and fears of being rejected and discriminated against are evidence of the persistent nature of AIDS-related stigma in communities and households. AIDS-related stigma remains one of the barriers to curb the further spread of the disease amongst people who are aware of their HIV-positive status [21]. Thus, in the South African context, AIDS-related stigma poses a major challenge to the efficacy of risk reduction interventions for PLWHA and other risk groups. 

Related to stigma was the extent of *othering* of the disease, with notions of the virus spread by being promiscuous (especially for women), gay or black:

*“My mother had AIDS and they said that she was sleeping around and she was drinking a lot *…* I think in the rural areas they still have that mentality that the person was sleeping around *…*She has AIDS, she has AIDS, she is a slut.”* (FGD—HIV-positive African women)

*“The idea that HIV and AIDS is a punishment from God I think runs through all religions, and that you're being punished for leading a promiscuous life or immoral life or whatever, so that is also a dominant theme in the Muslim community.”* (KI—Community Muslim AIDS support group)

*“It's supposedly a black and a gay disease.”* (FGD—HIV-positive African men and women)

Perceptions of men who have sex with men (MSM), heterosexual women (as compared to men), other cultures and races other than one's own being responsible for the spread of the HI virus all appeared to contribute towards the further stigmatisation of PLWHA. Blaming “the other” for the spread of the virus leads to a denial of one's own vulnerability to HIV infection [22]. Denial of the vulnerability to HIV infection was also apparent among participants in this study:

*“I mean I know a lot of people like that, they never face any of the issues and they die because of, because they can't get medical help because they don't want to acknowledge they've got HIV you know.”* (FGD—HIV-positive white gay men)

*“Every time when people die “No, he had TB, he or she”. No, I tell them, even my brother, he passed away, I saw him, I know the symptoms, and I went to his doctor to tell him I want to know his status because I'm also HIV-positive, and his doctor told me he was HIV-positive, but at home they denied that my brother was HIV-positive.” *(KI—National PLWHA organisation)

In addition, people tended to distance themselves from HIV, giving it other names. They also did not talk openly about HIV in the community and this fostered stigma:

*“What is “this thing”? They do not want to understand that I have HIV, they do not want to say it, “I have this thing” *…* because of the stigma that is attached to HIV in the townships it becomes so terrible, and it is making our work very difficult.” *(KI—National AIDS organisation)

In line with these perceptions, reports of AIDS-related stigma in the Western Cape province of South Africa is such that HIV/AIDS is called “*ulwazi*” which means “that thing” [23]. “That thing” not only implies that there is no cure, but also suggests that it is a stigmatised illness that cannot be referred to by name [24].

Gender also plays a strong role in experiences of stigma. AIDS-related stigma for women is intensified because of their subordinate role in society [25, 26]. Interviews with KIs in this study revealed that AIDS-related stigma is a barrier for women accessing free voluntary counselling and testing (VCT) and prevention of mother-to-child transmission (PMTCT) services. For example, mothers who are HIV-positive find it difficult to comply with medical advice to formula feed, because of the fear of having their HIV status exposed:

*“When we tell them they have to choose as to what they are going to do with their baby, ‘Are you going to breastfeed your baby or are you going to bottle feed?' You will hear they will say ‘I can't give my baby *[formula feed brand]. *Why?' Because people in the location know that if you give your baby *[formula feed brand] *you are HIV-positive.” *(KI—PMTCT organisation)

On the other hand, once diagnosed HIV-positive, men avoided seeking help, treatment, or support, for fear of stigma. Such behaviour was seen as related to the male “macho” culture of needing to be seen to be strong, while HIV was still seen as largely a “female thing,” and thus it was mainly women who attended support groups:

*“With men we've been having little success, but we've been trying to get them on board, and it's a battle to get men involved, but also trying to make men aware that it's not only a women's issue, and they also need to be involved and come on board, so that's a bit of a struggle.” *(KI—Community Muslim AIDS support group)

*“Most of the time its females, because males, I don't know whether they are scared, most of the support groups are female. Maybe the man, if he has a big problem or if he was sick for a long time, very, very sick, so he thinks now he must go to the support group *…* I think maybe it's part of our culture, because I know actually men, they don't cry out, maybe they keep it inside, most of them they prefer to keep it inside than to talk to someone.” *(KI—National PLWHA organisation)

Furthermore, for MSM, sexual orientation could add to existing stigma attached to being HIV-positive:

*“Gay men have been told for generations, because of homophobia and prejudice, that their sex is bad, you understand? So the element of shame around HIV infection interfaces very directly with a diagnosis of HIV-positive in gay men *…* we talk about the stigma of being HIV-positive, but before we label them as HIV-positive, they are human beings and socially they are labelled for being gay, so you need to address that one before you can address the HIV stigma.” *(KI—Gay support NGO)

Interestingly, according to KIs and focus group participants, the levels of stigma and discrimination were decreasing and attitudes were shifting toward greater acceptance of PLWHA in local communities:

*“I think there are a lot of changes now within the community. You have to accept it *[your HIV-positive status]* firstly, and then others will too. People don't isolate themselves like they used to.”* (FGD—HIV-positive African women)

*“In K* [local predominantly African community]* it's different than the other areas, because we've been talking about HIV from 1999 *…* so they *[PLWHA]* don't really encounter more difficulties than a person who's HIV-negative.”* (KI—ARV counsellor coordinator)

#### 3.1.2. Disclosure

Decisions about HIV disclosure are dependent on perceived AIDS-related stigma [27]. In this study, closely linked to stigma were issues of disclosure. Due to the persistent nature of AIDS-related stigma group participants expressed fears of disclosure that might lead to rejection by their families or partners, or losing their jobs:

*“For me it was fear of rejection that somebody should find out that I'm positive, so for a long time I kept it to myself.”* (FGD—HIV-positive white gay men)

*“The second thing is the disclosure, ‘I can't disclose to my partner because he's going to throw me out of the house, I can't disclose to my family'.” *(KI—AIDS training coordinator)

Focus group participants described other manifestations of discrimination including being isolated and forced to use separate kitchen utensils, and keeping to “their own room”:

*“At the time that I told them *[my family]* they said they must buy me gloves so that when I cook food I must wear them *…* they said that it would be much better if I moved out of their house. I didn't say anything, I took my belongings and I went to my own place.”* (FGD—HIV-positive African women)

*“That thing of being discriminated against at your own home, just because they have found out that you are HIV-positive, they provide you with your own spoon and dish, they give you your own room, and there is no-one who wishes to come to your room, you sleep alone.”* (FGD—HIV-positive African men)

The insistence that the person living with HIV have their “own dish, spoon … everything that you are using … must be different” demonstrates the imagined danger, which the person living with HIV poses to the rest of the family. Inaccurate beliefs about HIV transmission can lead to more fear and discrimination, which can further stigmatise people living with HIV [28]. In a study done in South African communities, results showed that participants who held traditional beliefs about the causes of AIDS were more likely to stigmatise people living with HIV [29]. Similar experiences of discrimination and AIDS-related stigma were reported in a study conducted in an urban informal settlement in Cape Town [30]. Manifestations of such discrimination were also reported in the first nationally based HIV prevalence study in South Africa in which 26% of respondents revealed that they were unwilling to share a meal with an HIV-positive person, while nearly 18% indicated that they would not sleep in the same room with someone with AIDS [31].

Participants identified additional problems for women who disclosed their status to spouses and family members, as they could face divorce, being ejected from their home, or even subjected to violence:

*“When my partner discovered that he is also HIV-positive, he wanted to kill us both, he was devastated.”* (FGD—HIV-positive mixed race, men and women)

This was particularly problematic where the woman was financially dependent on a male partner:

*“The first thing you hear is ‘I am not working, maybe I am staying with this particular somebody, I don't have a place to stay, I'm staying at his place or his shack or whatever, if I told him that I'm HIV- positive, I'm out of his place, so I won't have anything to eat, and I'm not working'.” *(KI—NGO counsellor coordinator)

Also HIV-positive MSM can find themselves vulnerable to rejection from family and friends when they have not disclosed their sexual preference:

*“90% of the men here haven't even told their family members they were gay, never mind that they are positive *…* wow, you haven't even told your family that you are gay, or your friends, so how can you open up and tell them that you're positive?” *(KI—Workplace AIDS education organisation)

There was general agreement among participants in the study that it was necessary to disclose one's status to a potential sex partner. However, across focus groups participants talked of their sex partners' negative reactions and unwillingness to get tested:

*“When I found out that I am HIV-positive, I told my partner, my husband that I'm HIV-positive, then he said he is fine, he won't go for a test *…* and now he's not here, he's in Joburg with my children.”* (FGD—HIV-positive mixed race men and women)

*“In some other cases our boyfriends run away, for instance mine ran away and I was having a baby child *…* and my boyfriend ran away when the child was six months.”* (FGD—HIV-positive African women)

VCT is the cornerstone of HIV prevention in South Africa, and is also necessary for directing HIV-infected people to risk reduction counselling, treatment, and care services [32, 33]. However stigmatising views of PLWHA may jeopardise people's willingness to get tested [21]. Interestingly in this study, stories of acceptance by families and partners, and recognition of the value of disclosure in empowering and uniting PLWHA were also evident:

*“I told my husband ‘Look here, I have AIDS', he just sat down and said ‘Oh, no!' *…* I was just crying and then he supported me *…* we went to the clinic together, and then he said he wants to know everything that is going on, what he is supposed to do.” *(KI—PMTCT organisation)

*“What we find also that with disclosure comes a lot of empowerment, as well as where people disclose and they feel good about themselves, they think they're making a difference in other people's lives, they go to clinics and give talks.” *(KI—Community Muslim AIDS support group)

Positive attitudes and perceptions towards PLWHA were also reported in the second South African national sero-prevalence study in 2005 [34].

#### 3.1.3. Risky Behaviour

Several concerns were raised relating to risk factors for HIV infection, such as revenge sex by those infected:

*“Then you get the one that tests positive again, and just sleeps with anybody and says ‘I'm going to infect as many people as I can' *…* when someone is out for revenge.”* (KI—Community AIDS support organisation)

The role of alcohol and drugs in risk-taking behaviours also became evident in KI interviews and group discussions:

*“When you're dealing with white urban moffies [35], one of the things that you need to look at is recreational drug use for instance.” *(KI—Gay support NGO)

Gender violence was also mentioned as a potential risk factor for women:

*“We do recognise that there's a big problem in this community with violence against women and disempowerment of women *…* the women that we see don't actually have a choice as to what happens with their own sexuality and sexual patterns, and that's a problem.” *(KII—public hospital ARV medical officer)

These complex links between gender power relations, intimate partner violence, drug use especially among MSM [36, 37], and HIV/AIDS have been highlighted in a few local studies [38, 39].

Focus group participants felt that it was difficult to change risky behaviours. The use of condoms remained problematic, especially for men, with myths and notions of cultural barriers prevailing. Disclosure for group participants was problematic, hence the negotiation of condom use also became challenging, particularly if HIV-positive status had not been disclosed:

*“When you tell him to use a condom, they ask ‘which means you don't trust me/or are you sleeping around?' ” *(FGD—HIV-positive mixed race group—HIV-positive mixed race woman)

*“Because of our culture, because to us black people it's a new thing that thing of using condoms *…* our partners they just told themselves that they will not use condoms, and he does not think about you.”* (FGD—HIV-positive African women)

#### 3.1.4. Socioeconomic Issues

Focus group participants spoke of current socioeconomic circumstances as a challenge to living positively with the virus. Likewise, KIs expressed concern that PLWHA might have other, more pressing, problems that they faced on a daily basis (like unemployment, poverty, domestic violence), so that in contexts where concerns regarding survival from day to day were paramount, HIV status could be considered secondary:

*“I would say we need to look at social upliftment, that's a very important aspect, because sometimes the core of your problem starts at social level.”* (FGD—HIV-positive mixed race men and women)

*“People come because they are HIV-positive, they come to the support group, but many times the HIV status is almost secondary in the support group, it's like a whole lot of other issues that come out about poverty and about unemployment, about domestic violence, about child support, just everything else but the HIV status.”* (KII—Community Muslim AIDS support group)

*“Thabo *[Mbeki, SA president] *is dead spot on, poverty is our biggest issue here, you start dealing with poverty, suddenly people will start having a will to live, but if you live in a shithole, how the hell are you going to have the will to live *…* even in the support groups in *[two local predominantly African areas] *it wasn't about that they were positive, it was about housing, food **all** other issues than HIV, so I mean, what I've learnt is that nine out of ten people have got so much shit on their plate before HIV arrives, that that's the stumbling block.” *(KII—Work-place AIDS education organisation)

These findings suggest that in the context of unemployment, poverty, and lower socioeconomic status, HIV status becomes a secondary concern to PLWHA. In a study conducted in a black township in Cape Town, findings revealed that AIDS was only one of the major social stressors threatening people living in everyday poverty [40].

### 3.2. Implications for the Adaptation of the Intervention Activities of Healthy Relationships

Data was used to ensure that all intervention materials were contextually relevant. We replaced the USA-developed movie clips with South African movie clips from popular local movies and television shows. A major innovation was to create storyboards using the movie clips for use in venues where there was no electricity and movie clips could therefore not be used.

Healthy Relationships makes extensive use of movie clips, story boards, and role plays in three of the five sessions. The purpose of making use of movie clips, story boards, and role plays is to elicit discussions about disclosure to family, friends, and sex partners and about risk reduction strategies. Therefore it is important that these activities are contextually relevant. Movie clips were selected based on the issues and challenges highlighted by focus group participants and KIs. For example in this study data revealed that AIDS-related stigma are not eradicated that it remains persistent, however, there was also a sense among KIs and focus group participants that it has decreased substantively. The data also revealed that AIDS-related stigma manifested differently across cultural groups. Closely related to this was the extent to which “othering” is still occurring. On the basis of this, a clip of PLWHA from different cultural backgrounds speaking of their experiences of living with HIV and of AIDS-related stigma and their triumph over the management of the disease was selected for inclusion in the intervention package. This was also translated onto storyboards for the sessions that focus on disclosure. “Othering” was dealt with by the inclusion of an HIV/AIDS information session that dispelled some of the myths about HIV transmission that persisted in certain cultural groups.

Secondly, a dominant outcome of experiences of stigma was reluctance to disclose HIV-positive status, for fear of being rejected by one's spouse, sexual partner, or family members; losing one's job; being ostracised in the community. However, among focus group participants and KIs, the benefits of disclosure were also recognised. These findings confirm the need for issues of disclosure to be a central component of a risk reduction intervention for PLWHA. Previous studies have emphasised that disclosure of HIV-positive status is an integral part of behaviour modification [41]. According to research, three behaviour domains identified as problematic for people living with HIV are condom use, negotiation of safer sex practices, and disclosure [42]. Condom use and negotiation of safer sex practices involve the issue of whether to disclose one's serostatus to potential sex partners or not. Therefore, in the context of prevention of the further spread of HIV disclosure of HIV-positive status is important.

With regards to the adaptation of Healthy Relationships, the intervention activities and disclosure content have to be sensitive to differences of experience across local communities (e.g., Muslims), in different contexts (e.g., the work place), and for different groups (e.g., those with same sex sexual orientations). Formative research of this nature provides an indication for positive prevention interventions as to which social and sexual contexts motivate disclosure decisions. What also needs to be taken into consideration for the adaption of the intervention is the encouraging fact that some participants indicated that disclosure was not always followed by rejection, but that it could also lead to support. 

A key issue emerging from the qualitative interviews was the central role that gender plays in dealing with HIV-positive status. This manifested in a number of ways: firstly, HIV-positive women were often stigmatised as being promiscuous; secondly, disclosure rendered women especially vulnerable to rejection by partners and family, or being ejected from their home, or even being subjected to violence; thirdly, given prevailing gender power relations it was difficult for women to negotiate condom use with their male partners; finally, men were seen as reluctant to own the issue, by going for testing, using condoms, or attending support groups. Hence, sensitivity to gender relations during intervention adaptation is an important aspect. Gender relations should also be dealt with in relation to condom use specifically where women found it difficult to negotiate condom use in certain cultures. The dominant theme of gender power relations where women remained vulnerable and subordinate to their male counterparts manifested in women being subjected to rejection and outright violence by their partners. During the five day training, group facilitators were made aware of these gender relations in dealing with HIV-positive status. In addition to this we also compiled a resource list of available support centres for women who experienced gender based violence, and provided a list of nearest HIV testing centres for their male partners who are reluctant to test. Lastly, we also made female condoms available to the women who participated in our study, but acknowledged that this was a short-term solution.

Even though Healthy Relationships is primarily an HIV risk reduction intervention for PLWHA another important consideration arising out of the KI interviews and FGDs was the imperative to take the broader socioeconomic context of the lives of PLWHA into account. Specific issues such as psychosocial needs and the community context need to be considered. More recently, positive prevention has been criticized for its narrow focus on risk reduction, instead of following a more holistic approach that includes issues of nutrition and other aspects that impact on living positively for the person living with HIV. These findings demonstrate the importance of taking the broader socioeconomic context of PLWHA into consideration in the design of risk reduction interventions. In view of this, with regards to design and implementation of risk reduction interventions for PLWHA, collaboration with all service providers for PLWHA is integral to the implementation of the intervention in the next phase of the study [43].

## 4. Conclusions

The data presented in this study was the first part of a larger research project to evaluate the efficacy of a culturally adapted Healthy Relationships risk reduction intervention in Southern Africa. The next phase of the study will determine the feasibility of the implementation of Healthy Relationships and will assess the acceptability of the intervention protocol to both group facilitators and participants. Hence, this study did not examine whether some of the concepts used in Healthy Relationships (e.g., assertive listening) is appropriate for use in South African context. This will be determined in the next phase of the study.

Nevertheless, this qualitative study of the challenges faced by PLWHA has highlighted the ongoing role of AIDS-related stigma in local communities, as well as the impact of such stigma on decisions to disclose HIV status and negotiate safer sex. Our findings also pointed to the broader social context of factors like poverty and prevailing gender relations which may contribute to infection and reinfection among PLWHA and their partners. These findings thus indicate the strong need for HIV risk reduction interventions with those who know that they are living with HIV/AIDS. Moreover, the study has identified important issues which need to be considered in the adaptation but also the development of locally relevant interventions for PLWHA, and suggests some specific areas of focus for such programmes.
